# Supplementary material for: Causal Relationship between Aging and Anorexia Nervosa: A White-Matter-Microstructure-Mediated Mendelian Randomization Analysis
Source: Biomedicines. 2024 Aug 16;12(8):1874. doi: 10.3390/biomedicines12081874 (PMC11351342; doi:10.3390/biomedicines12081874)
Supplement: Supplementary file 1 [file biomedicines-12-01874-s001.zip › Supplementary Figure/Figure S4/Singe SNP plot of MR analysis for TL to ubm-b-1921.pdf]

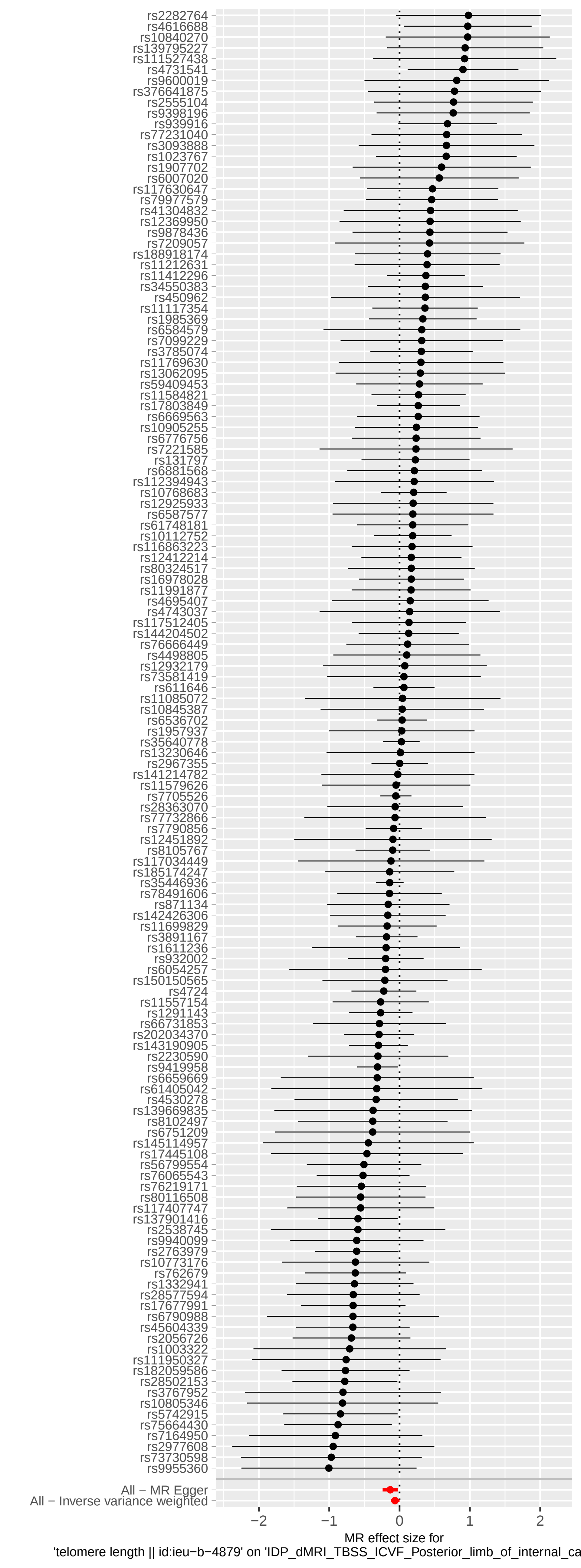
rs2282764
rs4616688
rs10840270
rs139795227
rs111527438
rs4731541
rs9600019
rs376641875
rs2555104
rs9398196
rs939916
rs77231040
rs3093888
rs1023767
rs1907702
rs6007020
rs117630647
rs79977579
rs41304832
rs12369950
rs9878436
rs7209057
rs188918174
rs11212631
rs11412296
rs34550383
rs450962
rs11117354
rs1985369
rs6584579
rs7099229
rs3785074
rs11769630
rs13062095
rs59409453
rs11584821
rs17803849
rs6669563
rs10905255
rs6776756
rs7221585
rs131797
rs6881568
rs112394943
rs10768683
rs12925933
rs6587577
rs61748181
rs10112752
rs116863223
rs12412214
rs80324517
rs16978028
rs11991877
rs4695407
rs4743037
rs117512405
rs144204502
rs76666449
rs4498805
rs12932179
rs73581419
rs611646
rs11085072
rs10845387
rs6536702
rs1957937
rs35640778
rs13230646
rs2967355
rs141214782
rs11579626
rs7705526
rs28363070
rs77732866
rs7790856
rs12451892
rs8105767
rs117034449
rs185174247
rs35446936
rs78491606
rs871134
rs142426306
rs11699829
rs3891167
rs1611236
rs932002
rs6054257
rs150150565
rs4724
rs11557154
rs1291143
rs66731853
rs202034370
rs143190905
rs2230590
rs9419958
rs6659669
rs61405042
rs4530278
rs139669835
rs8102497
rs6751209
rs145114957
rs17445108
rs56799554
rs76065543
rs76219171
rs80116508
rs117407747
rs137901416
rs2538745
rs9940099
rs2763979
rs10773176
rs762679
rs1332941
rs28577594
rs17677991
rs6790988
rs45604339
rs2056726
rs1003322
rs111950327
rs182059586
rs28502153
rs3767952
rs10805346
rs5742915
rs75664430
rs7164950
rs2977608
rs73730598
rs9955360
All − MR Egger
All − Inverse variance weighted
−2
−1
0
1
2
MR effect size for
'telomere length || id:ieu−b−4879' on 'IDP_dMRI_TBSS_ICVF_Posterior_limb_of_internal_ca
